# Co-dependence of the neural and humoral pathways in the mechanism of remote ischemic conditioning

**DOI:** 10.1007/s00395-016-0568-z

**Published:** 2016-06-23

**Authors:** Jack M. J. Pickard, Sean M. Davidson, Derek J. Hausenloy, Derek M. Yellon

**Affiliations:** The Hatter Cardiovascular Institute, University College London, 67 Chenies Mews, London, WC1E 6HX UK; Cardiovascular and Metabolic Disorders Program, Duke-NUS Graduate Medical School, Singapore, Singapore; National Heart Research Institute Singapore, National Heart Centre Singapore, Singapore, Singapore

**Keywords:** Myocardial infarction, Remote ischemic conditioning, Autonomic nervous system, Vagus nerve, Intrinsic cardiac ganglia

## Abstract

The cardioprotection afforded by remote ischaemic conditioning (RIC) is mediated via a complex mechanism involving sensory afferent nerves, the vagus nerve, and release of a humoral blood-borne factor. However, it is unknown whether release of the protective factor depends on vagal activation or occurs independently. This study aimed to evaluate the co-dependence of the neural and humoral pathways of RIC, focussing on the vagus nerve and intrinsic cardiac ganglia. In the first study, anesthetised rats received bilateral cervical vagotomy or sham-surgery immediately prior to RIC (4 × 5 min limb ischemia–reperfusion) or sham-RIC. Venous blood plasma was dialysed across a 12–14 kDa membrane and dialysate perfused through a naïve-isolated rat heart prior to 35-min left anterior descending ischemia and 60-min reperfusion. In the second study, anesthetised rats received RIC (4 × 5-min limb ischemia–reperfusion) or control (sham-RIC). Dialysate was prepared and perfused through a naïve-isolated rat heart in the presence of the ganglionic blocker hexamethonium or muscarinic antagonist atropine, prior to ischemia–reperfusion as above. Dialysate collected from RIC-treated rats reduced infarct size in naïve rat hearts from 40.7 ± 6.3 to 23.7 ± 3.1 %, *p* < 0.05. Following bilateral cervical vagotomy, the protection of RIC dialysate was abrogated (42.2 ± 3.2 %, *p* < 0.05 vs RIC dialysate). In the second study, the administration of 50-μM hexamethonium (45.8 ± 2.5 %) or 100-nM atropine (36.5 ± 3.4 %) abrogated the dialysate-mediated protection. Release of a protective factor following RIC is dependent on prior activation of the vagus nerve. In addition, this factor appears to induce cardioprotection via recruitment of intrinsic cardiac ganglia.

**Electronic supplementary material:**

The online version of this article (doi:10.1007/s00395-016-0568-z) contains supplementary material, which is available to authorized users.

## Introduction

Remote ischemic conditioning describes the cardioprotective intervention, whereby brief periods of ischemia to an organ or tissue remote from the heart can protect the myocardium from a subsequent injurious ischemic insult [57]. This important discovery, further to that of classical ischemic conditioning [55], gave immediate potential for clinical translation. Indeed, since its inception in 1993, there have been many attempts to prove its efficacy in limiting injury associated with coronary artery bypass (CABG) surgery and acute myocardial infarction in patients, with varying levels of success [11, 14, 17, 30, 31, 53, 67, 71, 78, 80]. Perhaps a reason for this difficulty in translation is a significant gap in our understanding of the mechanism, in particular, how the protective message is communicated from the conditioned limb to the myocardium [33, 56]. This is the unique mechanistic trait of RIC, thus understanding how it occurs is of great importance, and we believe would aid more effective clinical translation.

The communication of RIC is classically thought to occur via one of two pathways. First, a humoral pathway whereby a factor is released in response to the conditioning stimulus which moves to the heart via the circulatory system and protects [20, 21, 46, 59, 66]. Second, a neural pathway whereby the conditioning stimulus induces sensory nerve firing which, via autonomic centres in the brainstem, leads to increased efferent autonomic tone to the heart, initiating cardioprotection [22, 26, 28, 47, 51]. These two pathways were originally thought of as mechanistically distinct; however, recent data have suggested that the two are interdependent. Indeed, release of the humoral mediator in response RIC is dependent on sensory innervation to the conditioned limb [37, 52, 60, 70]. The downstream target of sensory nerve activation leading to release of the humoral factor, however, is not clear. One possibility is the vagus nerve, which has been previously implicated in the RIC mechanism [22, 51]. Indeed, a recent study suggested vagal innervation to the gut was essential for the communication of RIC, perhaps, via release of a blood-borne factor [50]. However, although it suggests release of a factor is dependent on vagus nerve activation, these data presented does not absolutely prove this link as release of a humoral factor is not measured. This study investigates the hypothesis that the humoral mediator is released downstream of vagus nerve activation.

There exist within the heart intrinsic neural loops that are able to process sensory information from the myocardial milieu and modulate efferent autonomic output from intrinsic cardiac ganglia, without any necessary input from the central nervous system [3, 5–7]. These neural loops are at risk of destruction following myocardial infarction, and, indeed, remodel, such that the intrinsic cardiac nervous system (ICNS) no longer can function as normal [1, 29, 36, 58]. Vagus nerve stimulation has recently been demonstrated to ameliorate post-infarction remodelling of the ICNS [8]. Thus, given the putative importance of the vagus nerve in RIC, this study investigated the hypothesis that intrinsic cardiac ganglia are recruited by the humoral mediator of RIC as part of the transduction of protection within the myocardium.

## Materials and methods

### Materials

Hexamethonium (Sigma-Aldrich, Missouri, USA) was employed as a neuronal nicotinic acetylcholine receptor (nAChR) antagonist. Given it has affinity to the muscarinic M2 receptor above 100 μM [24], a dose of 50 µM was used for this study to aid specificity at nAChRs within cardiac ganglia. Atropine, a muscarinic acetylcholine receptor (mAChR) antagonist, was used at a dose of 100 nM. Although often used at micromolar doses in the literature, atropine has a high affinity for mAChRs (*K*_d_ = 0.36 nM [72]). Thus, 100 nM was chosen, which has recently been demonstrated to effectively antagonise muscarinic agonism in isolated hearts [38].

### Animals and ethical statement

All animals received humane care in accordance with the United Kingdom (Scientific Procedures) Act of 1986. Male Sprague–Dawley (SD) rats were bred at a central animal unit in University College London and were used at a weight of 250–300 g throughout the study.

### In vivo procedure: donor rat

Male SD rats were anesthetised via an upper-left quadrant intraperitoneal injection of 20 % w/v sodium pentobarbitone (Animalcare, York, UK) at a dose of 0.05 ml/100 g + 0.05 ml. Once anesthesia was reached, confirmed via loss of pedal reflex, the animal was secured in a supine position on a heat mat. The rat was intubated via optic light trans-illumination of the trachea, using a modified 16G, 1.7 × 51 mm Abbocath-T intravenous cannula (B. Braun, PA, USA). This cannula was connected to either a PhysioSuite (Kent Scientific, CT, USA) or Small Animal Ventilator (Harvard Apparatus, Kent, UK), and ventilated with air supplemented with 0.5 l/min oxygen. Finally, core body temperature was maintained at 37.0 ± 0.5 °C via a rectal probe feeding back to a heat pad.

### Model characterisation

**Fig. 1:**
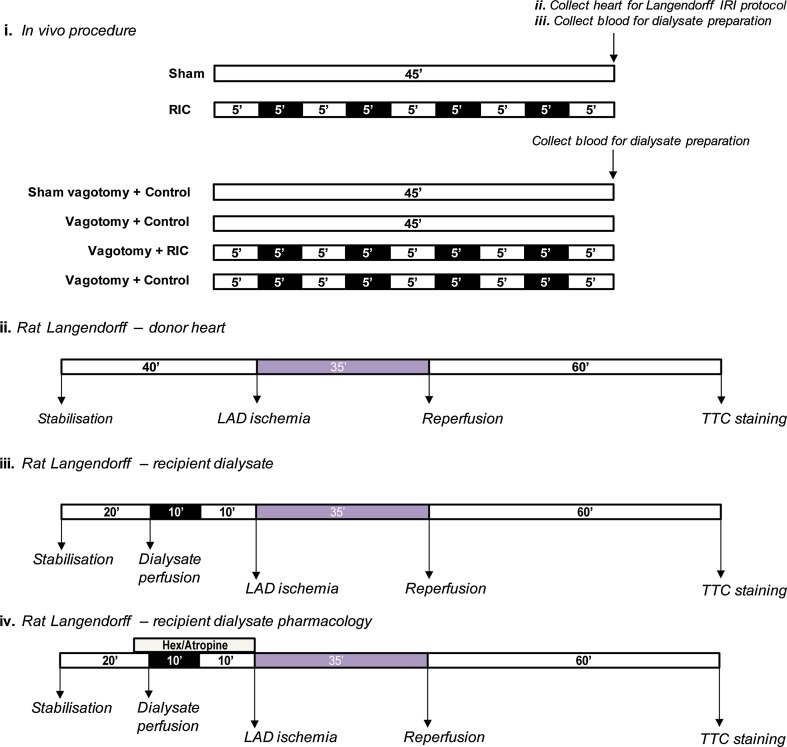
Design of experimental protocols: *i* displays in vivo procedures. Anaesthetised rats were subjected to either RIC (4 × 5 min hindlimb ischaemia/reperfusion) or sham protocols. Bilateral cervical vagotomy or sham-surgery immediately prior to either RIC (4 × 5-min limb ischaemia–reperfusion) or sham-RIC. *ii* At the end of this protocol, the heart was excised and perfused on a Langendorff rig before being subjected to a 35-min region ischaemia and 60-min reperfusion. *iii* Following the in vivo procedure, 9-ml blood was sampled and the plasma dialysed across a 12–14-kDa membrane. The dialysate was perfused through a naïve-isolated rat heart for 10 min prior to a regional ischaemia–reperfusion protocol, as described above. *iv* Isolated hearts were treated with either hexamethonium (50 μM) or atropine (100 nM) for 5 min prior to and the duration of dialysate perfusion/washout. Following each Langendorff experiment, infarct size (IS) was determined using TTC staining

Animals had a small cuff placed around their left hindlimb and were randomised to receive either (1) RIC protocol, consisting of 4 × 5-min cuff inflation to 200 mmHg with intermittent 5-min deflations, or, (2) sham-RIC protocol, consisting of a corresponding time period with no cuff inflation (see Fig. 1i). Following the procedure, a clamshell thoracotomy was performed and the heart was excised and perfused on a Langendorff apparatus, using the same method as described below. Following a 20-min period of stabilisation, hearts were subjected to a 35-min LAD ischemia and subsequent 60-min reperfusion [25] (Fig. 1ii). In a separate group of rats, blood was sampled via cardiac puncture following the protocol and prepared for dialysis as described below.

### Dialysate preparation

At the end of the protocol, ~9 ml of venous blood was harvested via right ventricular puncture, following clamshell thoracotomy, using a 1.5-in 19G Terumo needle (Egham, UK). Samples were immediately centrifuged at 1600*g* for 20 min at 21 °C to obtain plasma, followed by 10,000*g* for 30 min at 21 °C to obtain 4 ml of platelet-free plasma. This was dialysed across a 12–14-kDa membrane (Spectra/Por, Spectrum Laboratories, Inc., Rancho Dominguez, CA, USA) into 200 ml of modified Krebs-Henseleit buffer (KHB) (118-mM NaCl, 4.7-mM KCl, 1.22-mM MgSO_4_·7H_2_O, 1.21-mM KH_2_PO_4_, and 1.84-mM CaCl_2_·2H_2_O) for 24 h at 4 °C. Prior to perfusion through the naïve heart, the dialysate was supplemented with 25-mM NaHCO_3_ and 11-mM d-glucose, gassed with 95 % O_2_ and 5 % CO_2_ and warmed to 37.5 °C.

### Naïve recipient hearts

Rats were anesthetised with an upper-left quadrant intraperitoneal injection of sodium pentobarbitone (60 mg/kg) (Animalcare, York, UK). Hearts were quickly excised via a clamshell thoracotomy and the aorta cannulated on a Langendorff apparatus and perfused with KHB (118-mM NaCl, 25-mM NaHCO_3_, 11-mM d-glucose, 4.7-mM KCl, 1.22-mM MgSO_4_·7H_2_O, 1.21-mM KH_2_PO_4_, and 1.84-mM CaCl_2_·2H_2_O (for detailed methods, see [9]). A fluid-filled latex balloon was inserted into the left ventricle to allow for measurement of functional parameters, including heart rate (HR) and left ventricular developed pressure (LVEDP). Coronary flow rate (CFR) was recorded throughout the protocol, and the temperature of the heart was maintained at 37.0 ± 0.5 °C. Finally, a 3–0 Mersilk suture (Ethicon, Edinburgh, UK) was inserted through the heart to surround the left anterior descending (LAD) coronary artery. Following a 20-min stabilisation, dialysate was perfused through the heart for 10 min with a subsequent 10-min washout before index ischemia. All hearts received a 35-min LAD ischemia, induced via reversible tightening of the suture, and 60-min reperfusion (Fig. 1iii). Although there is evidence that reperfusion can influence infarct size in mouse Langendorff [62], the use of 60-min reperfusion duration, as opposed to 120 min, in the rat Langendorff model does not influence the efficacy of triphenyl-tetrazolium (TTC) staining [25].

### Study 1: bilateral cervical vagotomy and RIC in rats

Anesthetised SD rats were randomly assigned to one of the four groups: (1) *Sham vagotomy* + *Control*, the left and right vagus nerves were isolated at the mid-cervical level, but not severed, and the rat received a sham-RIC protocol; (2) *Sham vagotomy* + *RIC*, the same as group 1; however, the rat was subjected to RIC; (3) *Vagotomy* + *Control*, rats were subjected to bilateral cervical vagotomy and received a sham-RIC protocol; and (4) *Vagotomy* + *RIC*, rats were subjected to bilateral cervical vagotomy and received RIC (Fig. 1i). At the end of the protocol, blood was sampled via right ventricular puncture and dialysate prepared as described above. The dialysate was perfused through a naïve-isolated rat heart prior to IRI, also described above (Fig. 1iii).

### Study 2: intrinsic cardiac nerves and RIC in rats

Isolated hearts were randomly assigned to one of the following six groups: (1) *Control dialysate*, hearts received dialysate prepared from an in vivo rat following sham-RIC; (2) *RIC dialysate*, hearts received dialysate prepared from an in vivo rat following in vivo RIC; (3) *Control dialysate* + *Hexamethonium* (50 μM); (4) *RIC dialysate* + *Hexamethonium*; (5) *Control dialysate* + *Atropine* (*100* *nM*); and (6) *RIC dialysate* + *Atropine* (*100* *nM*) (Fig. 1iv). In groups 3–6, drug perfusion was initiated 5 min prior to and for the duration of dialysate treatment. The choices of drug concentration were chosen carefully, as described above. All hearts were perfused with the dialysate for 10 min, with a subsequent 10-min washout period, prior to IRI, as described above.

### Infarct size assessment

At the end of the reperfusion period, the LAD suture was re-tightened and 1 ml of 0.25 % Evans blue dye was perfused through the heart to delineate the area-at-risk of infarction. The hearts were then frozen at −20 °C before being sectioned into five-transverse slices and stained for viable tissue by immersion in 1 % triphenyl-tetrazolium chloride at 37 °C for 15 min. Following fixation in 10 % formalin for 24 h, the sections were digitally scanned to a computer for analysis. Analysis of infarct size (IS) as a proportion of area-at-risk (AAR) was calculated via planimetry using the ImageJ software (version 1.45, National Institutes of Health, USA). Infarct size was calculated a percentage of the area-at-risk (IS/AAR).

### Bicinchoninic acid (BCA) assay to measure dialysate and plasma protein concentration

Plasma or dialysate samples were assayed for protein concentration using the BCA assay method, as described previously [68]. Briefly, 5 μl of each sample is added to a 96-well plate, followed by 195 μl of a 1:50 mix of copper II sulphate and bicinchoninic acid. This is allowed to incubate for 30 min at 37 °C before being subjected to colorimetric analysis at 470 nm.

### Statistics

Data groups were first analysed for normality using the Kolmogorov–Smirnov test. Statistical differences between two groups were analysed using a student’s *t* test and more than two groups using a one-way analysis of variance (ANOVA) with Tukey’s multiple comparison post-test. Haemodynamic data in Figs. 4 and 5 were analysed using a two-way repeated measures ANOVA with Bonferroni’s post-test. All data are presented as mean ± standard error of the mean (SEM). Data groups were classed as significantly different with a *p* value less than 0.05. Notation of significance was as follows: * = *p* < 0.05, ** = *p* < 0.01, and *** = *p* < 0.001. Analysis was exclusively performed using GraphPad Prism version 5 for Windows (CA, USA).

## Results

### In vivo RIC induces significant cardioprotection

**Fig. 2:**
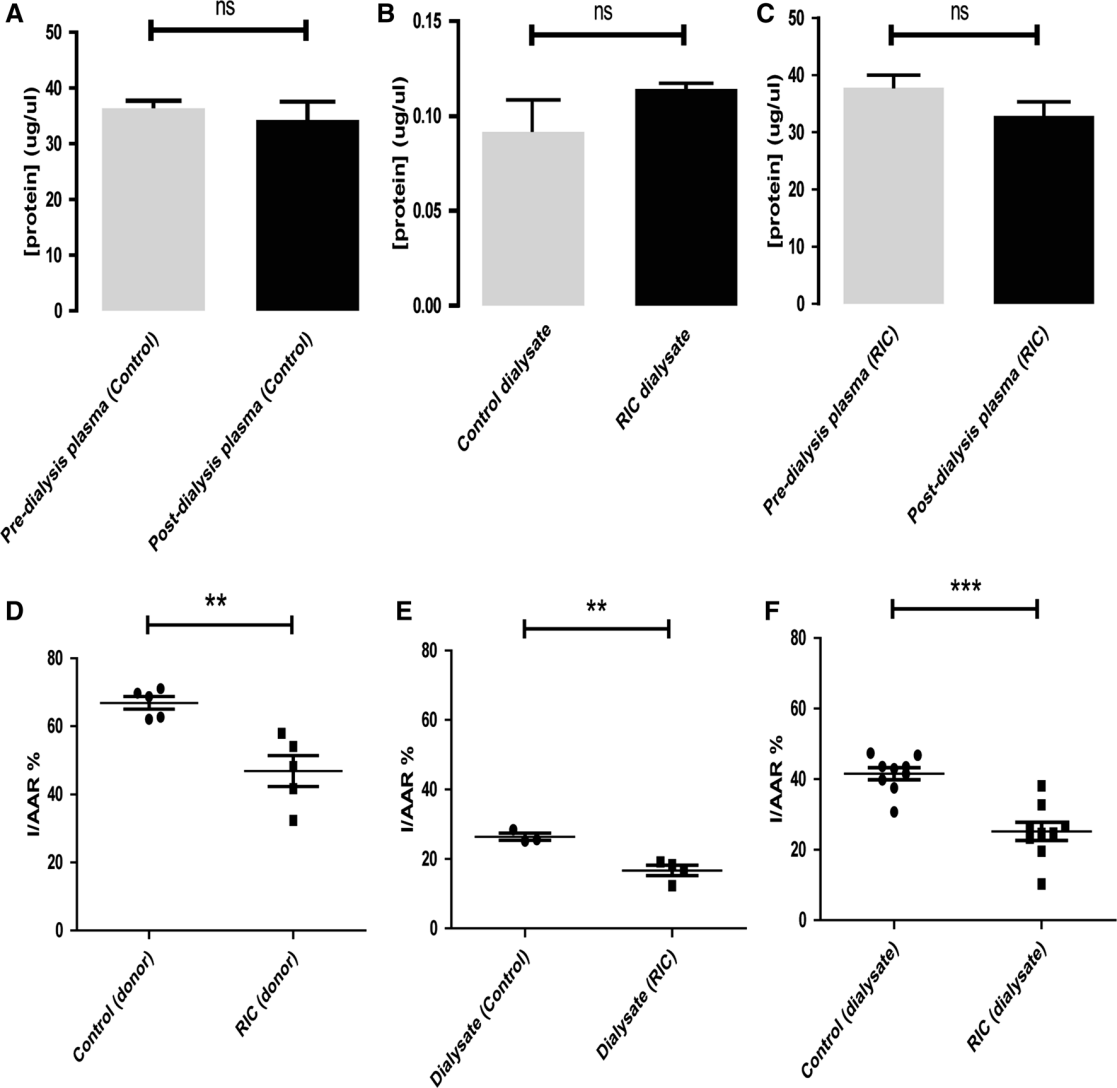
Characterisation of the rat dialysate model: **a**–**c** BCA protein assay was employed to measure protein concentration in the plasma following either RIC (4 × 5-min hindlimb ischaemia/reperfusion) or sham-surgery. No difference in plasma concentration was observed between pre- and post-dialysis groups, and no difference was observed following RIC relative to sham. Interestingly, a 400-fold reduction in protein concentration was observed within the dialysate when compared to plasma. **d** In vivo RIC or sham procedures preceded excision of the heart and perfusion on a Langendorff apparatus. These hearts were subjected to IRI, and those who had received RIC displayed reduced infarct size relative to control. **e** Dialysate was initially prepared using platelet-rich plasma. When perfused through a naïve-isolated heart, dialysate from both RIC and sham-treated rats gave a protected phenotype, although RIC dialysate still gave a significant additive protection. When platelets were removed from the plasma prior to dialysis (**f**), sham dialysate no longer gave a protective phenotype; however, RIC dialysate was able to significantly protect naïve hearts. Data were analysed via student’s *t* test, *n* = 6–8 per group, and expressed as mean ± SEM

The RIC protocol of 4 × 5-min hindlimb ischemia–reperfusion, induced via a small blood-pressure cuff, was effective at inducing cardioprotection in vivo, as demonstrated by a significantly reduced infarct size in hearts subjected to RIC relative to sham procedure (sham IS/AAR = 61.7 ± 1.9 % vs RIC IS/AAR = 46.9 ± 4.6 %, *p* < 0.05) (Fig. 2d).

### Model characterisation: RIC dialysate is cardioprotective to a naïve-isolated rat heart

The dialysate transfer model has previously only been described in the rabbit [37, 61, 70] and mouse [34]. Whilst plasma transfer from pig to rat heart has been proven [66], it was important to characterise the dialysate model in the rat. We initially took platelet-rich plasma from sham- or RIC-treated rats, dialysed across a 12–14-kDa membrane into a 50-fold volume of buffer, and perfused this through naïve-isolated rat hearts prior to ischemia–reperfusion injury (IRI). The RIC dialysate provided a significant protection to the naïve-isolated heart (sham dialysate IS/AAR = 26.4 ± 1.0 % vs RIC dialysate IS/AAR = 16.7 ± 1.5 %, *p* < 0.01, *n* = 6–8 per group) (Fig. 2e). However, the infarct size in isolated hearts that were perfused with the sham dialysate was unexpectedly small in comparison to typical control infarct sizes. In contrast, when platelets were removed from the plasma by centrifugation, infarct sizes were more typical, and RIC remained fully effective (sham dialysate IS/AAR = 41.5 ± 1.7 % vs RIC dialysate IS/AAR = 25.2 ± 2.6 %, *p* < 0.001, *n* = 6–8 per group) (Fig. 2f). We, therefore, used platelet-free plasma in further studies.

The concentration of protein within the plasma and dialysate did not differ between sham and RIC groups (sham = 0.09 ± 0.02 μg/μl vs RIC = 0.11 ± 0.002 μg/μl, *n* = 6/group, *p* > 0.05). There were no differences observed in the plasma protein concentration between RIC and sham groups before or after dialysis. With the 50-fold dilution and 12–14-kDa dialysis cutoff, a 400-fold decrease in protein concentration was observed between plasma and dialysate.

### Study 1: bilateral cervical vagotomy abolishes release of the humoral mediator following RIC

**Fig. 3:**
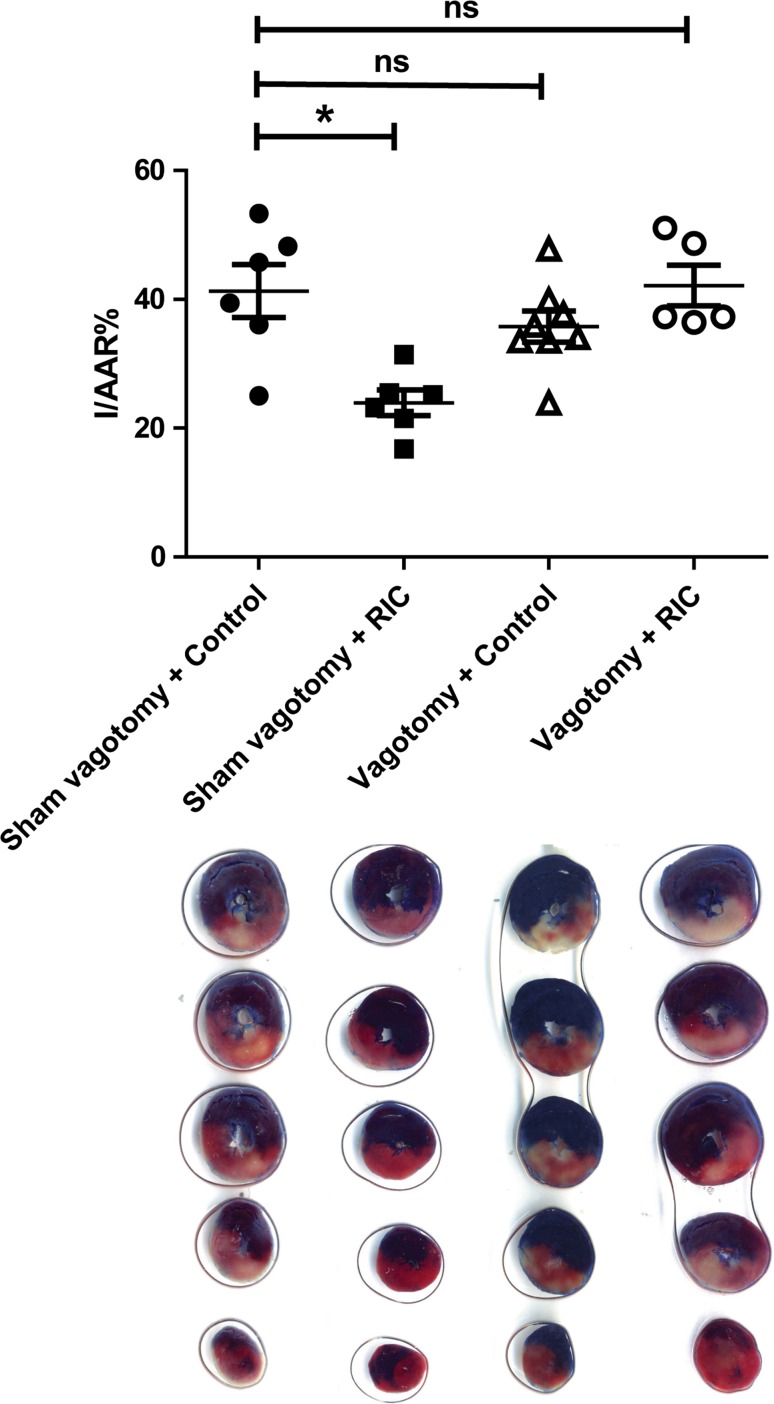
Bilateral cervical vagotomy abrogates dialysate-mediated protection of naïve-isolated rat hearts: the chart displays left ventricular infarct size as a proportion of the area-at-risk. Dialysate was prepared following in vivo vagotomy or sham-surgery matched with either RIC or sham protocols. This was perfused through a naïve-isolated heart prior to IRI. Sham vagotomy did not influence the ability of RIC dialysate to induce cardioprotection in the naïve heart. Bilateral cervical vagotomy, however, abrogated RIC dialysate protection in the naïve heart, suggesting that release of the blood-borne humoral mediator was inhibited. Data were analysed via one-way ANOVA with Tukey’s post-hoc test, and presented as mean ± SEM, with 6–8 animals per group

Sham surgical vagotomy did not influence the efficacy of RIC dialysate to protect a naïve, isolated rat heart (control dialysate from sham-surgery rat: IS/AAR = 40.7 ± 6.3 % vs RIC dialysate from sham-surgery rat IS/AAR = 23.7 ± 3.1, *p* < 0.05) (Fig. 3). When the vagus nerve was sectioned bilaterally at the cervical level, RIC dialysate no longer protected the naïve-isolated heart (control dialysate from vagotomised rat: IS/AAR = 31.4 ± 2.4 % vs RIC dialysate from vagotomised rat: IS/AAR = 42.2 ± 3.2 %, *p* < 0.05 vs sham-surgery RIC dialysate) (Fig. 3).

The hemodynamic data for the naïve-isolated hearts that received dialysate are shown in supplementary Fig. 5. Measurements were taken at the beginning of stabilisation, 5 min into the index ischemia and at the end of 60-min reperfusion. Those hearts treated with RIC dialysate did not demonstrate improved functional recovery as measured by coronary flow rate (CFR), left ventricular developed pressure (LVEDP), and heart rate (HR). In addition, dialysate prepared following bilateral cervical vagotomy did not affect the functional recovery when perfused through a naïve-isolated heart.

### Study 2: the humoral RIC mediator exerts protection via intrinsic cardiac nerves

**Fig. 4:**
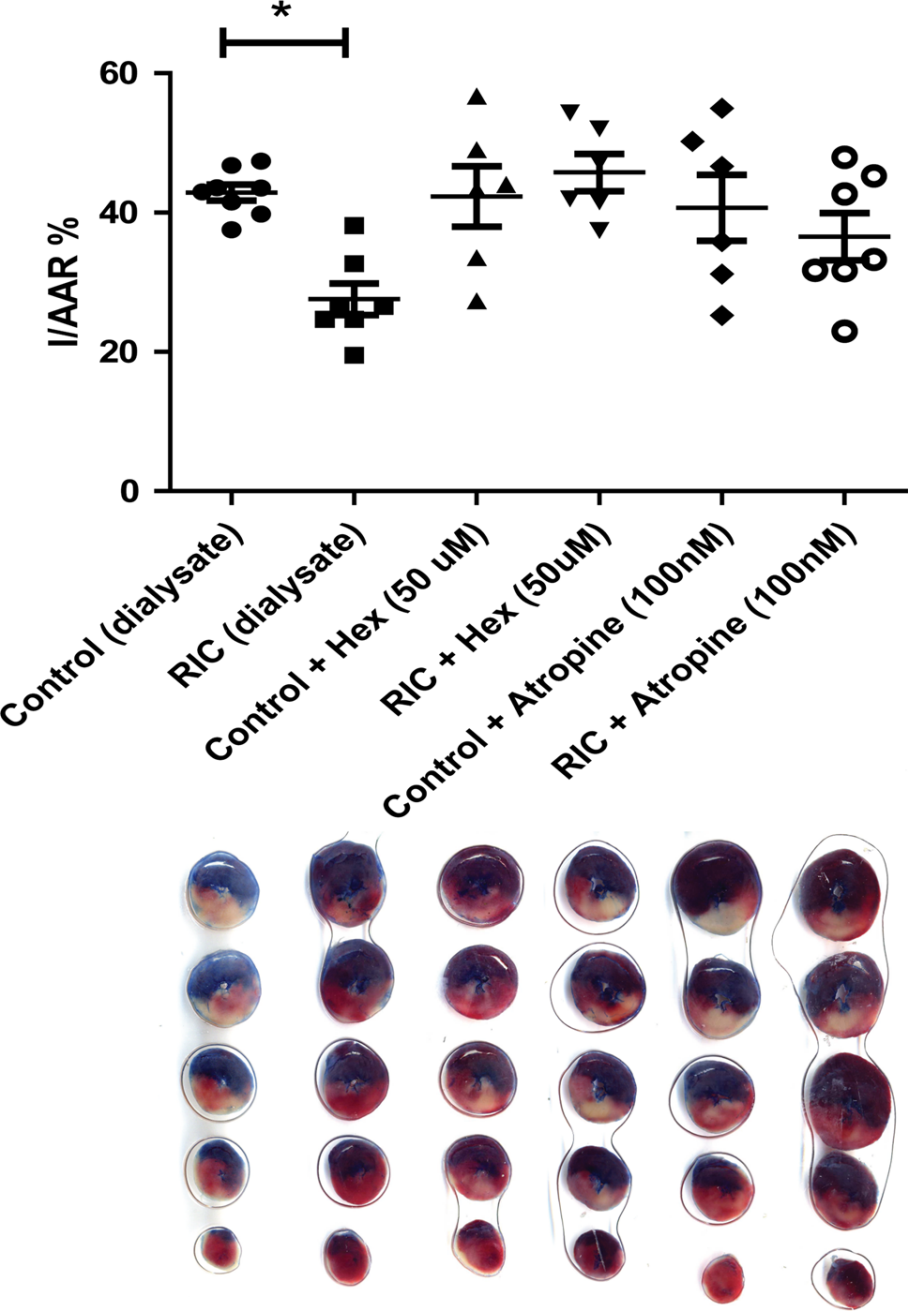
Hexamethonium and atropine abrogate dialysate-mediated cardioprotection: isolated rat hearts were perfused with dialysate prepared following in vivo RIC or sham procedures. RIC dialysate significantly protected the naïve heart from IRI relative to sham. When the naïve heart was pre-treated with either the ganglionic antagonist hexamethonium (50 μM) or the muscarinic antagonist atropine (100 nM) abrogated this protection. Data expressed as mean ± SEM, *n* = 6–8 per group

Figure 4 displays the infarct size chart and corresponding functional data from naïve-isolated rat hearts subjected to dialysate in the presence or absence of either the ganglionic blocker hexamethonium or the muscarinic antagonist atropine. Those hearts treated with RIC dialysate in the absence of either drug-induced powerful cardioprotection (sham dialysate IS/AAR = 40.1 ± 1.2 vs RIC dialysate IS/AAR = 27.6 ± 2.3, *p* < 0.05). In the presence of hexamethonium (50 μM), RIC dialysate was no longer able to protect naïve hearts (sham dialysate + Hex IS/AAR = 42.3 ± 4.3 % vs RIC dialysate + Hex IS/AAR = 45.8 ± 2.7 %, *p* > 0.05 vs sham dialysate). The muscarinic antagonist atropine (100 nM) also abrogated RIC dialysate-mediated cardioprotection (sham dialysate + atropine IS/AAR = 40.7 ± 4.8 % vs RIC dialysate + atropine IS/AAR = 36.5 ± 3.4 %, *p* > 0.05 vs sham dialysate) (Fig. 4).

The hemodynamic data again indicated that RIC dialysate did not significantly influence functional recovery of naïve hearts relative to control (Supp. Fig. 6). In addition, neither hexamethonium nor atropine affected functional recovery of the isolated hearts.

## Discussion

Our study elucidates two novel aspects to the mechanism of RIC communication. First, release of the blood-borne humoral mediator is dependent on prior activation of the vagus nerve. Second, the humoral mediator exerts cardioprotection in the myocardium in part via the recruitment of intrinsic cardiac ganglia. Whilst the importance of the vagus nerve in RIC has been previously reported [22, 23, 50, 51], this study is the first to prove its involvement in the release of the humoral mediator. We have demonstrated that a plasma dialysate generated following in vivo RIC in rats is able to significantly protect a naïve-isolated rat heart from ischemia–reperfusion injury. Previous studies have prepared dialysate from an in vivo rabbit model [52, 60, 70] or, indeed, humans [34, 37, 64]. These used a 20-fold dilution gradient into the dialysate, which is 2.5-fold lower than the 50-fold dilution used in this study. This demonstrates both the conserved nature of the RIC mechanism across several mammalian species and the remarkable potency of cardioprotection offered by the factor(s). The interesting observation that platelet-rich plasma generates a cardioprotective dialysate is most likely a systematic effect. Platelets are thought to degranulate at low temperatures [54], and when activated are known to release several cardioprotective molecules that are smaller than 12–14 kDa, for example, stromal-derived factor 1α [13, 15, 16, 20, 49]. The plasma is dialysed at 4 °C, therefore, during this process, the platelets may degranulate and release these cardioprotective factors. However, this was not investigated in this study, and instead, platelet-free plasma was used for dialysis in the two subsequent experiments. Finally, recent evidence from our laboratory has suggested that the duration of reperfusion may influence the magnitude of protection induced by pre-conditioning [62], perhaps due to limitations of the TTC method of infarct analysis. The evidence in rat Langendorff studies, however, indicates that one can observe powerful cardioprotection at both 60 min [79] and 120 min [32] of reperfusion. Thus, the use of 60 min reperfusion can give an accurate account of infarct size within the confines of the TTC staining method.

Vagus nerve stimulation has been reported in the literature to offer significant cardioprotection from ischemia–reperfusion injury [22, 51, 65, 73]. Indeed, an important study by Mastitskaya et al. demonstrated that activation of a particular group of pre-ganglionic parasympathetic neurones, in the dorsal vagal motor nucleus (DVMN), was sufficient to induce powerful cardioprotection in vivo [51]. In a subsequent and very elegant experiment, the DVMN was genetically silenced using the allatostatin method [51, 76]. This process abrogated RIC-mediated cardioprotection, indicating that this group of pre-ganglionic parasympathetic neurones is fundamental for the communication of the protective message from the conditioned limb to the myocardium. Moreover, a recent study from the same group indicates vagal innervation to the stomach, and gut is responsible for RIC communication, suggesting the release of a blood-borne mediator following vagal recruitment [50]. The literature, however, is not fully in agreement with these data [23]. Donato and colleagues demonstrated that cervical but not sub-diaphragmatic vagotomy abrogated RIC, suggesting direct cardiac vagal innervation to be key for RIC communication. The absence of a sham sub-diaphragmatic vagotomy group, however, calls the result into question given the huge abdominal trauma associated with the abdominal surgery. In addition, release of a humoral cardioprotective mediator downstream of vagus nerve activation has not been demonstrated in the literature. The key result from our study, therefore, is that release of the humoral RIC mediator is dependent on prior activation of the vagus nerve. We further conclude that the humoral factor is released from a region of the body innervated by the vagus nerve below the cervical level. Whether this factor is released following non-cardiac vagal innervation, however, is not clear.

**Fig. 5:**
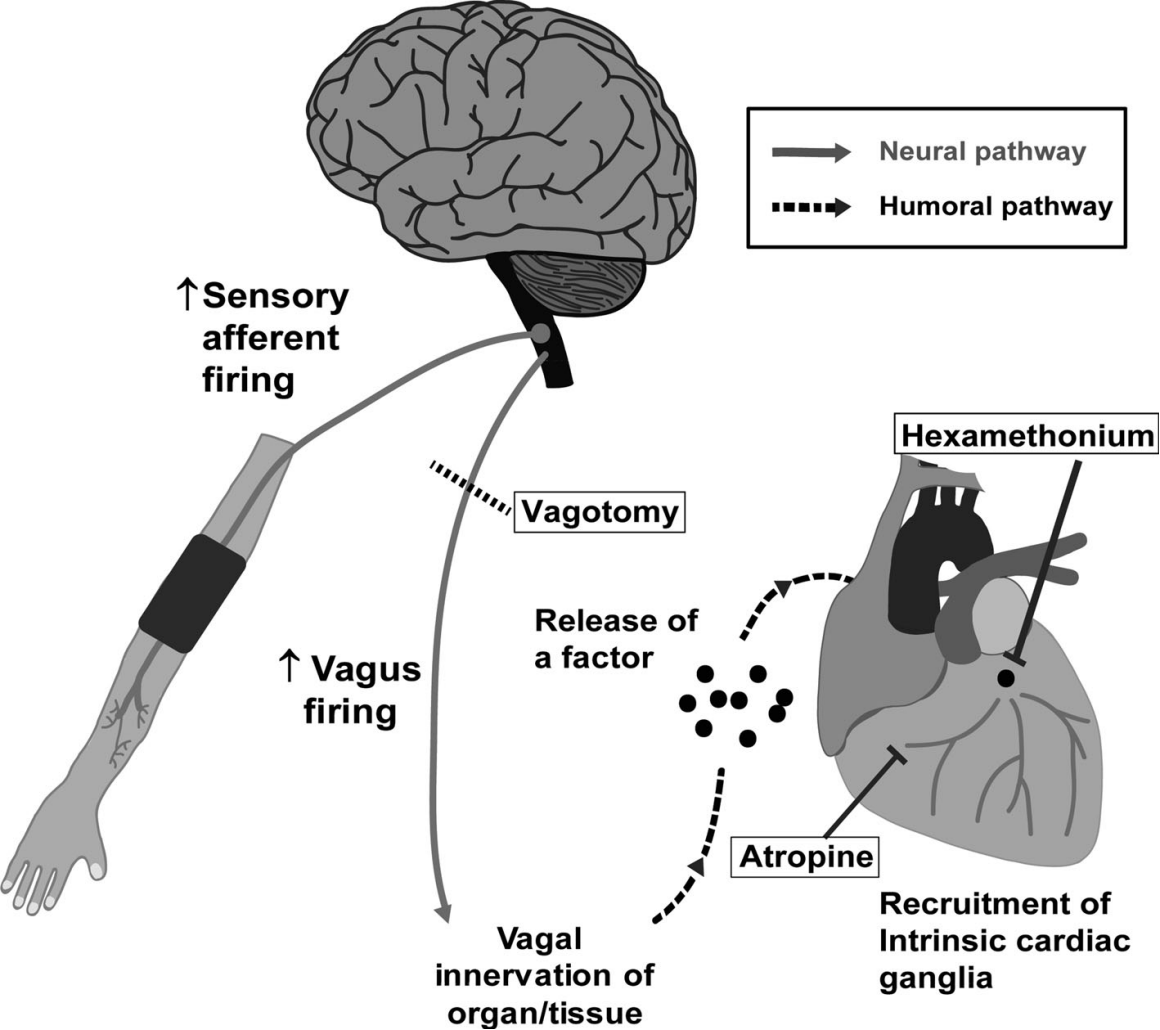
Schematic of proposed mechanism of RIC communication (figure adapted from [56]): serial inflations and deflations of a cuff around the upper limb will activate sensory afferent nerves [70]. These, in turn, will convey their message to autonomic regions of the brainstem, leading to increased systemic efferent vagal tone. The vagus nerve will innervate an organ remote from the heart, which induces release of a dialysable cardioprotective factor less than 12–14 kDa in size. This factor will move to the heart via the blood and induce a protective phenotype within the myocardium, in part via the recruitment of intrinsic cardiac ganglia

Release of the humoral mediator following RIC is dependent on an intact sensory innervation to the conditioned limb [37, 70]. In addition, two important studies demonstrated that direct femoral nerve stimulation, topical application of capsaicin or, indeed, transcutaneous electrical nerve stimulation generated a plasma dialysate that was able to protect a naïve heart from ischemia–reperfusion injury [52, 61]. These data suggest that the sensory afferent nerve is the sole means of communication from the conditioned limb, and that the factor is released downstream of nerve stimulation. Our study, perhaps, adds to the model by suggesting that the vagus nerve is the link between sensory nerve activation in the limb and release of the humoral mediator (Fig. 5).

Growing evidence suggests that cardiac neural control is hierarchical. Indeed, sensory information from the heart can be received by: (1) central nervous control from medullary autonomic centres in the brain, which provide information to the heart via autonomic efferent pre-ganglionic neurons; (2) intrathoracic extracardiac ganglia; (3) intrinsic cardiac ganglia [2–6, 35, 41, 48]. Intrinsic cardiac ganglia are able to process sensory information from the myocardium and directly activate efferent post-ganglionic nerve firing from intrinsic cardiac ganglia, thus neural control of the heart can occur without any extracardiac input [7, 41, 42]. The Langendorff perfused isolated heart is traditionally thought of as a denervated preparation; however, in light of recent studies in the literature described above, as well as the results from this paper, it appears that intrinsic neural loops remain intact in the isolated heart and continue to play an important role in its function and ability to withstand ischemia–reperfusion injury.

Transmission of a sensory message in a pre-ganglionic synapse within intrinsic cardiac ganglia is governed via the release of acetylcholine into the synaptic cleft, which will bind to and activate nicotinic acetylcholine receptors (nAChR), on the post-ganglionic nerve, causing a depolarisation and initiation of the nerve impulse [10, 19, 27]. Hexamethonium will antagonise the nAChR, thus preventing transmission of information at the ganglia. The observation, therefore, that hexamethonium abrogates dialysate-mediated protection suggests that the humoral factor recruits intrinsic cardiac ganglia as an essential process in the induction of cardioprotection. Muscarinic acetylcholine receptors (mAChR) are present primarily on the sarcolemma of cardiomyocytes [12]. They respond to acetylcholine released from parasympathetic post-ganglionic neurons, which innervate the ventricles [18, 39, 63, 74]. Indeed, their activation has been previously implicated in cardioprotection, with exogenous acetylcholine inducing powerful protection to isolated perfused rat hearts via an Akt-dependent mechanism [45]. Thus, the observation that atropine, a mAChR antagonist, abrogates dialysate-mediated cardioprotection suggests that the humoral factor induces increased intrinsic post-ganglionic parasympathetic nerve outflow. Previous evidence has demonstrated that hexamethonium and atropine abrogate in vivo RIC in rats [22, 26, 51], although the literature is not in full agreement [77]. However, it was not clear from these studies which ganglia are important for the transmission of the RIC protective message. In addition, evidence that the endothelium can be protected by RIC discounts the importance of intrinsic nerves in responding to the humoral mediator [43]. However, the protection offered to endothelium by RIC may be obtained via a different mechanism relative to the myocardium. Our data suggest that the intrinsic cardiac ganglia play an important role in this setting.

One interpretation of these data is that vagus nerve stimulation may be sufficient to evoke all of the protection conferred by RIC. In the literature, chronic vagal nerve stimulation failed to ameliorate cardiac remodelling or functional capacity in heart failure patients [81]. However, whether vagus nerve stimulation can protect the heart from acute myocardial infarction (AMI) in the clinic is unknown. Perhaps, the cuff inflation used currently to induce RIC could be replaced with non-invasive stimulation of the vagus nerve [69]. A recent clinical study demonstrated that the anesthetic propofol impedes the ability of RIC to protect the heart [44]. Propofol is known to be inhibitory to vagus nerve activity [75], thus suggesting the anesthetic prevents the communication of RIC at the level of the parasympathetic centres in the brainstem. Perhaps, RIC should be given, while the patient remains conscious, prior to administration of the anesthetic. Secondly, vagal tone is thought to depreciate with age [40]. Therefore, given the high average age of patients who suffer AMI, their diminished vagal tone may reduce the efficacy of RIC. Further study is required to elucidate the effect of age and anesthetics on RIC, both in terms of vagal tone and function of intrinsic cardiac ganglia. In addition, investigation into which branch of the vagus nerve is responsible for inducing release of the factor will help reveal the site of its release and improve the chance of discovering its identity.

## Electronic supplementary material

Below is the link to the electronic supplementary material.

Supplementary material 1 (DOCX 81 kb)

Supplementary material 2 (TIFF 9588 kb)

Supplementary material 3 (TIFF 7971 kb)

## Abbreviations

AAR: Area-at-risk
AMI: Acute myocardial infarction
BCA: Bicinchoninic acid
CABG: Coronary artery bypass surgery
CFR: Coronary flow rate
DVMN: Dorsal vagal motor nucleus
IRI: Ischemia–reperfusion injury
IS: Infarct size
LAD: Left anterior descending
LVEDP: Left ventricular end diastolic pressure
mAChR: Muscarinic acetylcholine receptor
nAChR: Nicotinic acetylcholine receptor
RIC: Remote ischemic conditioning
SD: Sprague–Dawley
