# Supplementary material for: Co-dependence of the neural and humoral pathways in the mechanism of remote ischemic conditioning
Source: Basic Res Cardiol. 2016 Jun 23;111:50. doi: 10.1007/s00395-016-0568-z (PMC4919370; doi:10.1007/s00395-016-0568-z)
Supplement: Supplementary file 1 — Supplementary material 1 (DOCX 81 kb) [file 395_2016_568_MOESM1_ESM.docx]

**Supplementary material**

**Supplementary figure 1. Risk region data for donor hearts:** *Area-at-risk (AAR) of infarction expressed as a percentage of the left ventricle. No difference in AAR was observed between control and RIC donor hearts.* *Data analysed by unpaired student’s t-test, p>0.05. Data expressed as mean ± SEM.*

**Supplementary figure 2. Risk region data for recipient hearts:** *Area-at-risk (AAR) of infarction expressed as a percentage of the left ventricle. No difference in AAR was observed between control and RIC donor hearts. Data analysed by unpaired student’s t-test, p>0.05. Data expressed as mean ± SEM.*

**Supplementary figure 3. Risk region data for vagotomy dialysate experiment.** *Area-at-risk (AAR) of infarction expressed as a percentage of the left ventricle. No difference in AAR was observed between groups. Data analysed via one-way ANOVA and expressed as mean ± SEM.*

**Supplementary figure 4. Risk region data for the intrinsic nerve pharmacology experiment.** *Area-at-risk (AAR) of infarction expressed as a percentage of the left ventricle. No difference in AAR was observed between groups. Data analysed via one-way ANOVA and expressed as mean ± SEM.*

**Supplementary figure 5. Haemodynamic data for bilateral cervical vagotomy experiment:** *Measurements were taken following 10min of stablilisation, 5min into the index ischaemia and at the end of reperfusion. (A) coronary flow rate is expressed as ml/min, no significant difference observed between groups. (B) left ventricular developed pressure measurement were obtained via insertion of a fluid-filled balloon into the left ventricle. RIC dialysate did not affect functional recovery of naïve-isolated hearts. (C) heart rate is expressed as beats-per-min, again there were no significant differences between the groups. Data expressed as mean±SEM, n=6-8 per group.*

**Supplementary figure 6. Haemodynamic data for the intrinsic cardiac ganglia experiment:** *Measurements were taken following 10min of stablilisation, 5min into the index ischaemia and at the end of reperfusion. (A) coronary flow rate is expressed as ml/min, no significant difference observed between groups. (B) left ventricular developed pressure measurement were obtained via insertion of a fluid-filled balloon into the left ventricle. RIC dialysate did not affect functional recovery of naïve-isolated hearts. (C) heart rate is expressed as beats-per-min, again there were no significant differences between the groups. Data expressed as mean±SEM, n=6-8 per group.*
