# Supplementary figures and images for: Co-dependence of the neural and humoral pathways in the mechanism of remote ischemic conditioning
Source: Basic Res Cardiol. 2016 Jun 23;111:50. doi: 10.1007/s00395-016-0568-z (PMC4919370; doi:10.1007/s00395-016-0568-z)

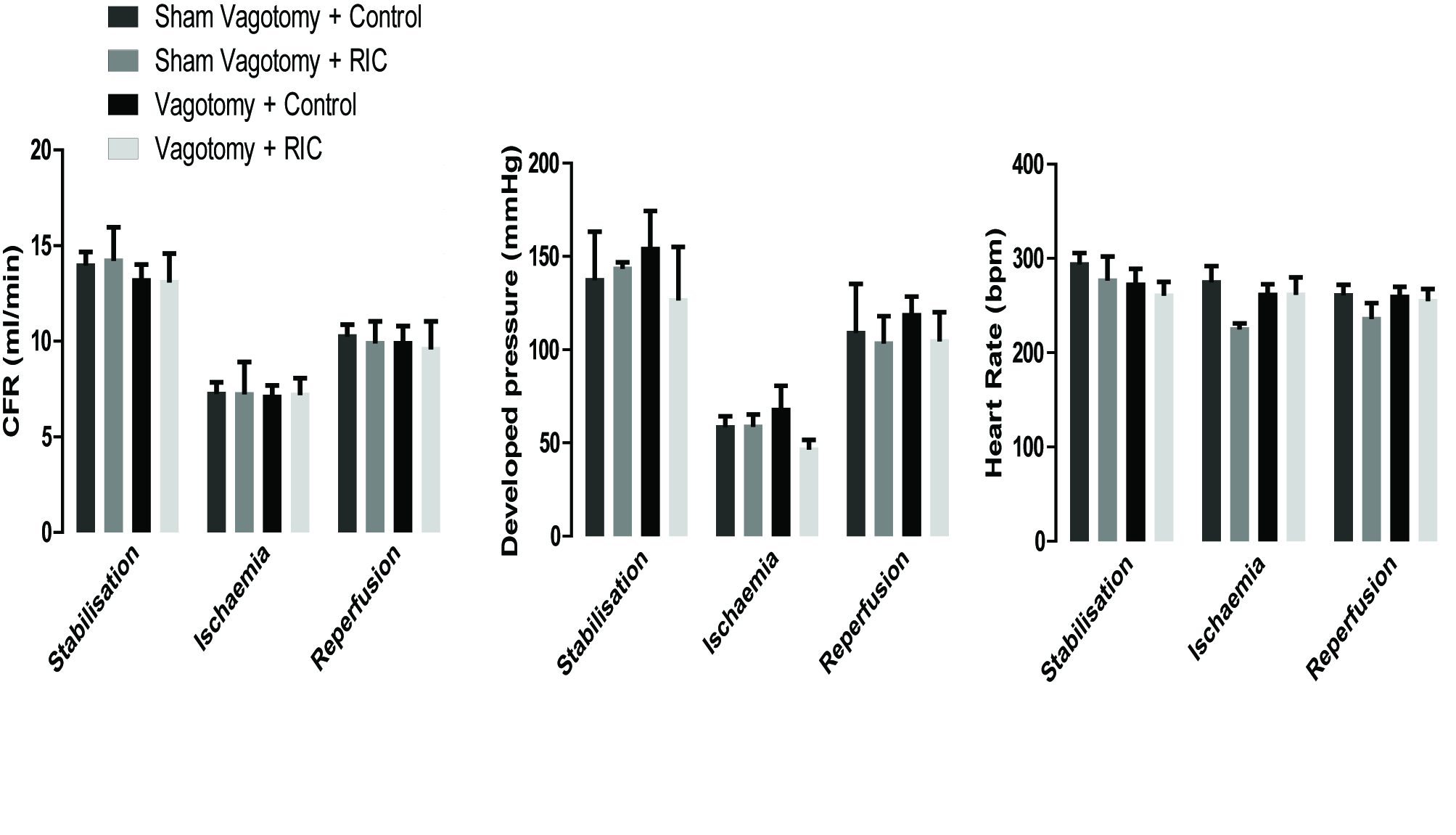

Supplement: Supplementary file 2 — Supplementary material 2 (TIFF 9588 kb) [file 395_2016_568_MOESM2_ESM.tif]

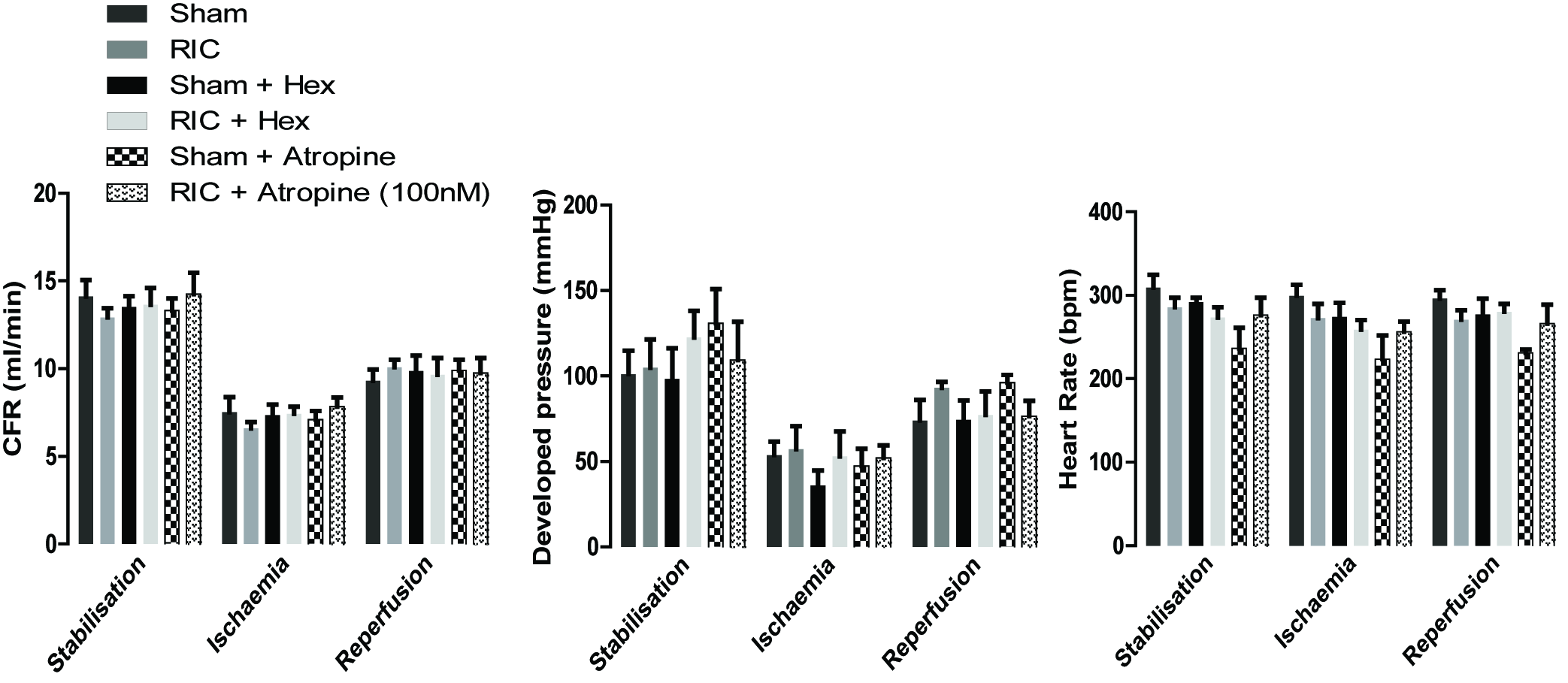

Supplement: Supplementary file 3 — Supplementary material 3 (TIFF 7971 kb) [file 395_2016_568_MOESM3_ESM.tif]
